# Gut Microbiome, Intestinal Permeability, and Tissue Bacteria in Metabolic Disease: Perpetrators or Bystanders?

**DOI:** 10.3390/nu12041082

**Published:** 2020-04-14

**Authors:** Rima M. Chakaroun, Lucas Massier, Peter Kovacs

**Affiliations:** Medical Department III—Endocrinology, Nephrology, Rheumatology, University of Leipzig Medical Center, 04103 Leipzig, Germany; lucas.massier@medizin.uni-leipzig.de (L.M.); peter.kovacs@medizin.uni-leipzig.de (P.K.)

**Keywords:** gut microbiome, intestinal permeability, leaky gut, extra-intestinal microbiome, obesity, insulin resistance, type 2 diabetes, metabolic disease

## Abstract

The emerging evidence on the interconnectedness between the gut microbiome and host metabolism has led to a paradigm shift in the study of metabolic diseases such as obesity and type 2 diabetes with implications on both underlying pathophysiology and potential treatment. Mounting preclinical and clinical evidence of gut microbiota shifts, increased intestinal permeability in metabolic disease, and the critical positioning of the intestinal barrier at the interface between environment and internal milieu have led to the rekindling of the “leaky gut” concept. Although increased circulation of surrogate markers and directly measurable intestinal permeability have been linked to increased systemic inflammation in metabolic disease, mechanistic models behind this phenomenon are underdeveloped. Given repeated observations of microorganisms in several tissues with congruent phylogenetic findings, we review current evidence on these unanticipated niches, focusing specifically on the interaction between gut permeability and intestinal as well as extra-intestinal bacteria and their joint contributions to systemic inflammation and metabolism. We further address limitations of current studies and suggest strategies drawing on standard techniques for permeability measurement, recent advancements in microbial culture independent techniques and computational methodologies to robustly develop these concepts, which may be of considerable value for the development of prevention and treatment strategies.

## 1. Introduction

The past few years have witnessed significant headway in the exploration of mechanisms underlying cardiometabolic diseases such as type 2 diabetes (T2D), obesity, and related cardiovascular comorbidities. Specifically, microbiome research has gained significant traction, placing it at the center stage of a plethora of noncommunicable diseases, ranging from autoimmune disorders of the gut such as inflammatory bowel disease (IBD) [1] to a wide range of psychological [2] and metabolic disorders [3]. Considering the essential contribution of bacteria to the great oxygenation event, the origins of life [4], and our persisting co-evolution, culminating in a microbiome surpassing our genetic arsenal by around 150 folds [5], it seems inevitable that our lives are intricately linked to that of the bacteria we share our bodies with. Epidemically expanding modern health ailments such as obesity, T2D, hypertension, and hyperlipidemia as well as cardiovascular disease originate—at least partially—from a derailed interaction between us, our microbiota, and our environment. Previous research has focused on the gut, which is the most heavily colonized area of the human body. Here, the extensive genetic arsenal of gut bacteria enables them to provide the host with a multitude of ecosystem services such as breaking down of otherwise indigestible polysaccharides, the production of vitamins, and the transformation of several xenobiotics, leading to the potential modulation of their therapeutic effects and their toxic potential [6]. For any of this to be possible, it is essential that the intestinal immune system is tolerant towards mutualistic or commensal microorganisms residing in the intestinal lumen and along its lining, whilst simultaneously keeping pathobionts in check and reducing incidents of putative bacterial translocation. This is achieved by a complex system of chemical and physical barriers as well as immunological lines of defense including immune cells and mediators, which development is extensively tailored by the gut microbiome itself [7].

While the crosstalk between the gut microbiome and peripheral organs such as the brain, liver, adipose tissue, muscle, and pancreas has emerged as a crucial factor for homeostatic systems, the search for channels of communication has led to the revival of the concept of “leaky gut”. This concept is based on the notion of translocation of whole bacteria, bacterial products such as metabolites, and bacterial wall components into the circulation and distant tissues, contributing to remote organ injury in metabolic disease. Although bacterial translocation is a physiological process crucial for host immunity [8], “pathological translocation” has been repeatedly evidenced, which is best exemplified in its clinical expression as spontaneous bacterial peritonitis (SBP). In this case, otherwise nonpathogenic intestinal bacteria are indivertibly found in mesenteric lymph nodes and ascites, leading to the observed local inflammation and clinical findings. The treatment comprises decontamination via oral antibiotics, which are usually only effective in the gut. Therefore, in order to treat the bacterial-induced inflammation in the peritoneum, it is imperative to treat the gut.

As subclinical inflammation has been evidenced in obesity leading to local and systemic insulin resistance and the development of cardiovascular disease, it is important to track similar mechanisms of action as seen in SBP in metabolically active organs and to understand the role of remote organ–bacteria interaction in the pathogenesis of noncommunicable diseases, which in turn could open novel avenues for prevention and therapy.

## 2. Gut Microbiome Shifts, Diet, and Intestinal Permeability in Metabolic Disease

Although growing beyond the scope of infectious disease for several decades, microbiome research has seen a remarkable boost in the last two decades thanks to the development of sequence-based techniques for the detection of microbes which escape traditional culture-based approaches. More importantly, there have been efforts to establish systematic approaches to decipher structure and function of the gut microbiome in health and disease with the Human Microbiome Project (HMP) [9] as well as the European MetaHit project [5], leading to a multitude of publications linking the gut microbiome with several factors detailed in excellent reviews on diet and microbiome modulation of health and disease [10,11,12,13], obesity [14], inflammation [15], metabolic disease [16,17], and cardiovascular disease [18] as well as comprehensive approaches on intertwined disease entities [19]. While the composition of the microbiome is highly dependent on age, sex, and ethnicity [20], several observational and experimental studies, discussed below, have used complementary approaches such as gut microbiota-directed interventions and suggest intimate links between microbiome shifts in metabolic disease as well as possible pathways shaping this relationship.

### 2.1. Compositional Gut Microbiota Shifts and Metabolic Disease Signatures

Early indications on the connection between gut microbiome and obesity stem from studies in both mouse models and humans [14,21,22,23,24]. Germ-free mice fed on a high-fat/ high-sugar diet are resistant to weight gain compared with conventionalized mice, even while consuming more calories overall. This was attributed to increased levels of phosphorylated AMP-activated protein kinase (AMPK) and its downstream targets of fatty acid oxidation in skeletal muscle and liver [22]. In addition, conventionalization of germ-free mice led to significant weight and fat gain despite unchanged food intake and normal energy consumption [21], further establishing the microbiome as a regulator of energy homeostasis.

Examination of identical twins, who were either concordant or discordant for obesity, showed that the development of obesity is related to microbiome shifts deviating from a “healthy core microbiome”, reflected by a decreased microbial diversity and altered representation of metabolic pathways [14]. Gut microbiome transfer from individuals with obesity into germ-free mice induced significant weight gain in the animals, which was not reproducible in those receiving microbiota transfers from lean individuals [24]. These data demonstrate a causal relationship between changes in the microbiome and the development of obesity with underlying mechanisms being similar across species. Moreover, reduced gut microbial diversity was associated with insulin resistance, increased circulating inflammation markers, and fatty liver. Patients with reduced microbiome diversity less frequently benefited from weight loss intervention in respect to improvement in markers of systemic inflammation, insulin resistance, and dyslipidemia [25], underscoring the importance of baseline microbiome signature in disease and intervention outcome.

Similarly, a bacterial signature is noted in T2D: In the largest study to date, reduced microbiome diversity as well as significant reduction in butyrate producers were evidenced in 345 Chinese patients with T2D, while the number of opportunistic pathogens increased [26]. Comparable results were demonstrated in 145 women with T2D in Sweden [27], where the gut microbiotal signature was more strongly correlated with T2D than with classic T2D risk parameters such as body weight, body mass index (BMI), waist circumference, or waist-to-hip ratio. Moreover, the continuous progression of T2D was characterized by an increasing loss of gut microbial diversity and specific taxonomic groups such as *Bifidobacteria* and *Verrucomicrobiae* [28]. A representative of the latter phylogenetic class is *Akkermansia muciniphila*, which has been associated with reduced weight and improved insulin sensitivity in mouse models [29] and humans [30] as well as with an improvement of glucose metabolism in humans upon Metformin initiation [31].

Evidence from cross-sectional studies was corroborated in intervention studies: Gut microbiome transfer from healthy subjects into patients with T2D resulted in a significant improvement in insulin sensitivity over a period of six weeks [32,33]. To this end, an increase in *Verrucomicrobiae* after fecal microbiome transfer (FMT) was associated with an antidiabetogenic effect, while increased Proteobacteria was associated with insulin resistance. Consistent with weight loss intervention, the improvement of insulin sensitivity was largely driven by baseline intestinal microbiota composition [33].

Similar observations were made on the relation of gut microbial diversity with nonalcoholic steatohepatitis (NASH) and nonalcoholic fatty liver disease (NAFLD) [34,35,36], although studies seem to be in less agreement on specific perpetrators. Echoing results emanating from the study of hypertension further demonstrated the additional involvement of reduced gut microbiome capacity for short-chain fatty acid production, especially butyrate, in blood pressure regulation [37,38,39]. The relationship between the gut microbiome and hypertension seems to be subject to further environmental control, as shown in the work of Wilck et al., who demonstrated that salt-responsive hypertension was associated with a depletion of *Lactobacilli* and that replenishing lost strains was associated with a reduced induction of Th17 Cells and reduction in hypertension [40].

### 2.2. Quantitative Gut Microbiome Shifts in Metabolic Disease: When Numbers Matter

Quantitative changes of the microbiome have also been reported in the literature for several metabolic diseases. Sabaté et al. reported a small intestine bacterial overgrowth (SIBO) prevalence of 17.1% in subjects with severe and morbid obesity [41]. In that particular study, SIBO seemed to be associated with severe hepatic steatosis. This has been underlined in several studies pointing rather to a significant association between SIBO and nonalcoholic fatty liver disease [42], whereas the association between obesity and the risk of SIBO has been deemed insufficiently proven according to meta-analyses [43]. The evidence for SIBO in diabetes (T1D and T2D) seems more substantiated [44] with prevalence of SIBO ranging anywhere between 11.6% and 60% depending on the test performed [42,45]. This association comes off as intuitive as SIBO has been traditionally linked, at least partly, to a decrease in intestinal motility [45], intestinal transit, and autonomic neuropathy [46]. Although evidence for a connection between SIBO and intestinal permeability measured via dual sugar absorption test has been established in NAFLD [47] as well as immunodeficiency diseases [48], it remains unclear whether SIBO leads to increased permeability or whether both conditions have their roots in an additional common denominator. While quantitative changes in the microbiome of the small intestine (as exemplified by SIBO) can be related to changes in the qualitative microbiome composition of the colon and with increased intestinal permeability, there is emerging evidence for important contribution of microbial quantity in the colon to health as well. Vadeputte et al. reported that quantification of bacterial profiles far bypasses compositionality analyses and revealed that the frequently reported trade-off between *Prevotella* and *Bacteroides* is an artificial product of data compositionality. The authors further associated the occurrence of low-cell-count enterotypes with Crohn’s disease, further underlining a relationship between intestinal bacterial load, microbiome composition, and inflammation [49].

### 2.3. Dietary Signals in the Crosstalk between Gut Microbiome and Intestinal Permeability

Quantitative and qualitative microbiome changes do not occur in pure isolation, and the interaction between diet and the gut microbiome on the one hand and impact of diet on intestinal permeability on the other have been the subjects of several recent extensive reviews and original work [50,51,52,53]. Effects on intestinal permeability are as one would expect for some nutrients, with several works converging on beneficial effects of peptides such as casein, vitamins such as vitamin D and retinol, polyphenols, and minerals such as zinc and on deleterious effects of alcohol and medium chains fatty acids (MCFA). Intriguingly, results of Eicosapentaenoic acid (EPA) and Docosahexaenoic acid (DHA) treatment on intestinal permeability diverged according to reporter cell system used [50]. Surprising effects are reported for specific amino acids such as glutamine and tryptophan, shown to decrease intestinal permeability via direct effects on tight junction expression [54,55], which was further corroborated in human trials, where supplementation of glutamine reduced intestinal permeability and endotoxin levels in burn victims and was associated with a shorter hospital stay [56]. Effects of amino acid modulation of intestinal inflammation via the microbiota are also evident with a recent work showing that arginine supplementation inhibited *Eggerthella lenta-*induced Th17 activation and subsequent colitis [57] and that indole-3-propionate derived from gut microbial metabolism of tryptophan influences barrier integrity via pregnane X receptor and Toll like receptor 4 (TLR) signaling [58]. However, increased meat protein intake has been suggested to contribute to incidence and severity of inflammatory bowel disease with colonic meat fermentation and release of toxic compounds such as ammonia, phenols, and branched-chain amino acids [59], mediating intestinal barrier impairment in a microbiota-dependent manner [60]. Similarly, the beneficial crosstalk between diet, microbiome, and intestinal barrier has been demonstrated for the microbial-mediated metabolism of dietary fibers and microbiota-accessible carbohydrates leading to an increased production of short chain fatty acids SCFA, modulating, in turn, intestinal mucosal immune barrier [61] and regulatory T-cell function such as Th17 [62].

In conjunction with evidence that environmental factors such as diet or other industrial food additives (i.e., salt [63,64]), as well a proinflammatory gut environment can lead to an increased intestinal permeability [65] and susceptibility to invasive pathobionts [66], and that inflammation per se can shift gut microbial composition [67], it is currently unclear whether inflammation is at the dispensing/receiving or both ends of gut microbiome shifts culminating in increased bacterial translocation.

Beyond the interplay between overall microbiome composition, inflammatory intestinal tone, and permeability, it is worth noting that some bacteria are likely to translocate more readily than others, this being related to their ability to forgo or debilitate host defense mechanisms. Gram-negative bacteria such as *E. coli* and other Enterobacteriaceae and Enterococci have been more often shown to translocate than other gut commensals [68,69]. Notwithstanding, it is conceivable that inflammation or metabolic stress can alter bacterial translocation rates either by increasing permeability to specific taxa or by stimulating active bacterial translocation by modulating bacterial mechanisms of pathogenicity [70].

## 3. Intestine’s Cerberus and the Leaky Gut

Although many associative studies have shown links between quantitative and qualitative changes in gut microbiome and metabolic disease, a closer look at the three main protective instances is warranted to discern mechanisms involved in connecting these events with metabolic pathologies.

### 3.1. Lymph Nodes and Immune Cells

The gut-associated lymphoid tissue (GALT) is the largest immunological organ of the human body. The innate immune system as a first line of defense entitles highly conserved recognition of microbe-associated molecular patterns (MAMPs), active on epithelial surfaces as well as within endosomes via TLRs or cytoplasmic NLR (nucleotide-binding domain, leucine-rich repeats proteins). Although perpetrating commensals are rapidly killed by macrophages, intestinal dendritic cells (iDCs) carry small amounts of live bacteria, which can then induce selective Immunoglobulin A (IgA) production in mesenteric lymph nodes (MLNs). These have been suggested to be at the center of bacterial translocation, as they restrict iDCs to the mucosal immune compartment. When MLNs are surgically removed, specific commensal IgA response is abrogated and bacterial systemic penetration is possible, as commensals can be retrieved from the spleens of animals without MLNs [71].

Activated B-cells and T-cells are recruited after bacterial translocation. B-cells produce commensal specific IgA which abrogates the translocation beyond the gut mucosa [72]. Mice deficient for TLR-dependent MyD88 on B-cells fail to induce an immunoglobulin response leading to systemic dissemination of commensal bacteria [73]. It is unclear whether this is associated with bacterial translocation in obesity and T2D, but there is accumulating evidence for B-cell dysfunction in obesity and T2D supporting a proinflammatory T-cells and a proinflammatory cytokine profile [74]. Likewise, the role of intestinal T-cells is not well defined in the emergence of obesity and T2D. Absence of gut-associated lymphoid T-cells leads to spontaneous translocation of commensal bacteria [75], and their suppression has been shown to enhance bacterial translocation in alcohol- and burn-related injuries [76]. Mechanisms underlying shifts in T-cells trafficking to the GALT, mesenteric lymph nodes, and intestine in response to enteric antigens have been implicated in the onset of chronic gut inflammation [77]. More interestingly, subjects with obesity and T2D displayed higher proportions of cytotoxic T-cells, activated T-helper cells and an impaired neutrophils function and T-cell response on challenge despite increased expression of activation markers [78]. This has been suggested as a possible mechanism responsible for increased prevalence of infection in subjects with T2D [79].

### 3.2. Secretory Compartment Including Mucus and IgA Antibodies

Bacteria close to the intestinal surface of the intestine are monitored and kept at a distance by several secretory elements encompassing antimicrobial proteins (AMPs) secreted mostly by Paneth cells after sensing of whole bacteria or lipopolysaccharides (LPS) via TLR-dependent MYD88 activation [80]. These include defensins, which have disruptive properties on microbial membranes, lectins, as well as bactericidal permeability-inducing proteins and resistin-like molecules. Subjects with obesity have lower AMPs including alpha-defensin as well as reduced lysozyme with signs of increased endoplasmic reticulum (ER) stress in paneth cells and an altered function, potentially contributing to the obesity-associated shift in microbiome or vice versa [81]. The mucus layer isolating the lumen from epithelial cells is furthermore actively involved in the intestinal homeostasis in health and disease as it encompasses AMPs produced in underlying cells. It also exhibits antibacterial activity [82] and has been shown to lose thickness after high-fat diet (HFD) [29]. Beyond fighting bacteria, mucus can feed specific types of bacteria such as *Akkermansia muciniphila*, which has been shown to reverse high-fat diet-induced metabolic disorders, including insulin resistance, and which has been shown to increase endocannabinoids tone controlling inflammation [29]. Moreover, bile acid disbalance seems to be relevant to metabolic regulation as bile acids modulate Glucagon-like Peptide 1 (GLP1) synthesis and secretion, which modulates food intake, intestinal motility, and insulin secretion and thus impacts obesity and T2D pathogenesis [83] as well as intestinal integrity [84]. In fact, bile reduction facilitates bacterial translocation and increases susceptibility to amplified translocation in response to endotoxin [85] or surgical trauma [86]. Another component of the secretory intestinal immune element is IgA antibodies, which can bind to bacteria, preventing their mucosal adherence as part of immune exclusion mechanisms [87]. HFD leads to a decrease in secretory IgA and high-fat diet-fed mice present with reduced glucose tolerance [88].

### 3.3. Intestinal Lining and Barrier Dysfunction

The mucosal lining of the gut consists of a single layer of cells constituting the mechanical component of the gut barrier. Bacterial translocation can occur through the paracellular route, which is regulated by tight junctions (TJs) or the transcellular route. Damage to the barrier function is reflected in increased permeability, which can be evidenced via dual sugar absorption tests. Specifically obesity has been associated with increased gut permeability [89], which correlates with insulin resistance (Homeostasis Model Assessment (HOMA) Index) positively [90] and is aggravated when liver injury is added to the equation [91]. Moreover weight loss retrieves gut permeability to normal range [91]. In a larger cohort, Genser et al. combined in and ex vivo investigations and showed that although subjects with obesity did not differ per se from lean subjects in fasting lactulose/mannitol urinary ratio or jejunal permeability in Ussing chambers tests, high jejunal permeability to small molecules (0.4 kDa) was related to increased systemic inflammation, suggesting at least a subtle dysfunction in gut permeability in obesity. This impairment was further exacerbated by lipid challenge in samples emanating from subjects with obesity as compared with lean subjects and was an independent explanatory variable for the presence of T2D [92]. Changes to the tight junction proteins from the claudin and zonula occludens family have also been observed with reduction in occludin and tricellulin [93] in obesity. This has been similarly shown in mice, where HFD induced a shift in claudin expression [94], as well as in hyperleptinemic db/db mice, which displayed a decreased intestinal resistance and a profound modification of the occludin and ZO-1 expression in their intestinal mucosa [95]. This observation was accompanied by higher circulating levels of tumor necrosis α (TNF-α) and interferon γ (INF-γ), which have independently been shown to increase occludins and the internalization of claudin-1 and -4 and to downregulate expression of claudin-1, causing increased permeability in colonic adenocarcinoma-derived T84 epithelial cell lines [93]. In the link between obesity and increased permeability, leptin could play a pivotal role. Leptin is not only increased after high fat intake [96] and associated with obesity [97], but also connected with obesity-linked mucosal intestinal inflammation [98]. Leptin treatment directly induces epithelial inflammation [99] and has been shown to increase proinflammatory activity of natural killer (NK) and CD8+ T-cells as well as TNFα-expressing cells in the gut, leading to autoimmune gut disease aggravation [100]. Proinflammatory macrophages are also shown to depend highly on glycolysis for their energy homeostasis [101]. Similarly Th17 cells with increased IL-17 production after leptin-induced anaerobic glycolysis are associated with neuroinflammation [102] as well as microbiota-modulated salt-responsive hypertension [40], allowing for a glimpse in the complexity of immunometabolism. There seems to be at least an adipose tissue–gut axis that is reflected in both adipose tissue and intestinal dysfunction. Whether the dysfunction-associated permeability leads to translocation of bacteria remains unclear. To this end, the transcellular pathway seems to be more relevant as transcytosis of living bacteria has been shown to occur independent of changes in paracellular permeability after metabolic and inflammatory stress to epithelial gut cells [70].

## 4. Breaking Down the Barriers: Markers of Bacterial Translocation

Beyond inflammation in the gut, obesity, insulin resistance, and subsequent metabolic diseases are frequently preceded and presumably precipitated by inflammation and dysfunction in visceral adipose tissue, liver, muscle, pancreas, and the brain. To connect intestinal health and microbiome profiles with metabolic health, most studies have focused on the study of surrogate parameters to evidence enteric permeability changes instead of performing functional tests (Table 1).

Cani et al. have coined the term “metabolic endotoxemia”, which describes an increased systemic exposure to bacterial LPS in obesity and insulin resistance [89]. The association between systemic bacterial inflammation and metabolic impairment stems from the early eighties, when it was shown that sepsis is associated with a reversible state of insulin resistance [103].

Until now, most research investigating bacterial translocation and metabolic disease has focused on LPS measurements, leading to a large body of evidence. LPS are components of the outer membrane of Gram-negative bacteria and are shed into the circulation mostly once a bacterium has been neutralized. Interestingly, fat intake has been associated with increased postprandial LPS, distributed in the circulation via formed chylomicrons [104], and direct administration of LPS induced systemic inflammation and insulin resistance [105]. The bridge between overnutrition and systemic inflammation was built, when it was shown that an 8 week long overfeeding intervention led to elevated endotoxin levels accompanied by insulin resistance [106]. Several large cohort studies have since then reported increased circulating levels of LPS in subjects with T2D, obesity, or/and metabolic disease [107,108,109]. The measurement of LPS Binding Protein (LBP), which modulates the biological activity of LPS, has been used interchangeably to convey a similar message [110]. Consistently, weight loss interventions as exemplified by bariatric surgery lead to improved glucose tolerance and overall metabolic improvement as well as weight loss alongside significant reductions in LPS and LBP levels [111,112], further supporting a direct link between postulated bacterial-induced inflammation and metabolic impairment. Although widely used, there are several limitations to the use of LPS as intestinal permeability markers. These are related in part to the fact that LPS measurement as a surrogate marker for “live” bacteria translocation is flawed by the necessity for bacterial death/lysis for LPS release. Several preanalytical issues including lack of sampling in pyrogen-free ware or the necessity for sample pretreatment to overcome low LPS recovery in the most widely used Limulus amoebocyte lysate test have contributed to a large range of LPS values reported in the literature [107,113]. Considering that LPS activity is actively modulated by host-derived protein binding and clearance [114], it becomes questionable that LPS measurement after pretreatment to overcome low recovery is reflective of in vivo conditions. This is supported by the fact that there is poor concordance between endotoxemia and Gram-negative bacteremia and that endotoxemia is detected in less than 50% of subjects with Gram-negative sepsis [115].

Bacterial products are not markers of bacterial translocation per se, but their shifts have been associated repeatedly with the concept of leaky gut and increased intestinal permeability as they are central to maintaining the intestinal barrier [116] and function [117]. Interestingly, physiological amounts of short-chain fatty acids have been shown to immediately support the colon barrier, which was shown via Ussing chambers [118]. Butyrate particularly has been shown to increase expression of tight junction proteins such as occludin, claudin, and zonula occludens [119] and to reduce bacterial translocation as measured via transepithelial resistance [120], and bacterial internalization via transmission electron microscopy after 24 h in preclinical models. This evidence, concomitant with reduction of butyrate producers in several metabolic diseases as mentioned above, could sustain the idea of bacterial products reflecting the status of the gut barrier. Nevertheless, these measurements have only been done with healthy controls as point of reference, making the use of bacterial products to prove the concept of leaky gut of secondary relevance.

Among other surrogate parameters, increased concentrations of circulating calprotectin are reported in T2D, which were associated with obesity, metabolic syndrome status, as well as myocardial infarction without being an independent predictor for cardiovascular disease (CVD) [121]. Calprotectin is a neutrophil-produced protein, which is most reflective of intestinal inflammation. There also seems to be a correlation between calprotectin levels, inflammation, and gut permeability, although this has been evidenced for irritable bowel syndrome and functional abdominal pain and might not specifically apply to metabolic diseases [122]. Similarly intestinal fatty acid-binding protein (IFABP) is uniquely located in mature enterocytes of the small intestine and shed into the circulation upon intestinal mucosal damage [123]. IFABP measurement in serum and urine has shown promising results in the context of intestinal ischemic injury in animal models and humans alike [123,124,125] and was significantly elevated in subjects with T2D [109], contributing to a permeability risk score used by the authors to describe elevated intestinal permeability in their T2D subgroup.

Markers such as endotoxin core antibodies (EndoCAb) have also been used and shown to be superior to endotoxin in one studied cohort, although only IgM antibodies were significantly different between lean subjects, patients with obesity, and T2D [126]. Considering that there lacks an absolute cut-off value for this specific method as well and that IgM and IgG kinetics are not well understood, it seems premature to recommend it as part of standard testing.

Other widely used surrogate markers such as zonulin, which increases intestinal permeability by dismantling intercellular intestinal tight junctions [127], are basically flawed by the fact that commercially available ELISAs are neither sensitive nor specific for zonulin itself [99].

A few studies have implemented functional testing of intestinal permeability using dual sugar absorption tests. There is robust data supporting the use of such tests [128]. Lactulose/mannitol test is the most extensively used test and has several clinical applications in the context of IBD or irritable bowel syndrome (IBS). The test is based on the measurement of two nonmetabolizable sugars, which are excreted into the urine after ingestion. The water-soluble monosaccharide mannitol passes the intestinal mucosa predominantly via transcellular uptake, whereas the larger disaccharide lactulose diffuses paracellularly and is usually held back by tight-junctions. The lactulose/mannitol index is therefore highly increased in intestinal inflammation and scenarios of increased intestinal permeability with disease relapse such as Crohn’s disease [129].

Preclinical methods such as Ussing chambers, histology, electron microscopy, and the gold standard method of measurement of transepithelial electrical resistance [128] are limited by the necessity for biopsies and/or other invasive tissue sampling and are therefore not readily available or applicable in the clinical setting, although the combination of in and ex vivo methods has proven highly complementary to evidence subtle impairments of intestinal permeability.

In summary, the line between increased gut permeability and bacterial translocation seems to be a hazy one, where sometimes neither increased gut permeability nor actual increased bacterial translocation is evidenced under the convenient title of leaky gut in the realm of permeability-induced metabolic impairment.

## 5. Bacterial Translocation and the Ominous T2D Octet

Bacterial signals can influence systemic inflammatory tone by increasing local inflammation in the gut, therefore leading to a widespread inflammatory response reaching several organs, or could potentially alter local signals in peripheral organs, leading to an overreaching inflammation and exponentiating insulin resistance. It is now recognized that several tissues play important roles in the advent of insulin resistance and related sequelae. Specifically, adipose tissue, the liver, muscle, gastrointestinal tract, the pancreas (β- and α- cells), kidney, and the brain collectively comprise DeFronzo’s ominous octet and contribute to the development of glucose intolerance and insulin resistance in T2D [138]. Considering the important therapeutic implications targeting each player, it seems noteworthy to address the effects of bacterial translocation on specific organs.

### 5.1. Adipose Tissue

Chronic inflammation of adipose tissue has been suggested as an underlying factor in obesity and insulin resistance. Kawano et al. have identified the colon as the first organ to respond to a HFD and contribute subsequently to adipose tissue inflammation and insulin resistance. HFD led to several morphologic as well as immunologic changes in the colon, including increased infiltration of macrophages accompanied by increased proinflammatory gene expression including *TNFA* and Interleukin 1B (*IL1B)*. Deletion of macrophages recruiting chemokine Ccl2 and its receptor in a knockout mouse model led to a decreased infiltration of colonic macrophages and inflammasome activation in the colon. This was associated with a decreased adipose tissue inflammation along with improved glucose and insulin tolerance compared with wild-type mice, albeit having similar body weight [139]. These results suggest that colonic inflammation leads to a remote control of adipose tissue inflammation and insulin resistance [140], possibly via systemically released inflammatory cytokines. Direct effects of bacterial signals have also been observed, although mostly in cell culture experiments, and direct stimulation of adipose tissue macrophages with LPS has been shown to induce adipose tissue fibrosis in a TLR4-dependent manner [141] and increase IL-6 and TNF-α, which could be abrogated upon NF-kB inhibition [142,143].

### 5.2. Liver

The body of work connecting liver disease with bacterial translocation is striking and has been elegantly reviewed in several works focusing either on steatohepatitis [144] or on liver cirrhosis [145]. Intestinal dysfunction related to increased permeability, quantitative microbiome shifts, or SIBO has been associated with insulin resistance and metabolic disease. In addition to associations between microbial diversity and NASH, LPS are evidently increased in the portal and/or systemic circulation in a multitude of chronic liver disorders [146]. Increased hepatic lipid accumulation is more frequent in increased fructose intake triggering hypertriglyceridemia, which may be in part brought about by an increased proinflammatory response associated with intestinal translocation of LPS. Fructose gavage led to increased hepatic TNFA expression and increased LPS levels in portal blood, which are reversed after antibiotic treatment [147]. Similarly, HFD led to increased plasma LPS [136], which in turn has been shown to induce foam cell formation [104] and increase NADPH activation in liver steatosis, contributing both to atherosclerosis and TLR4-mediated liver fibrosis [105]. In humans, dietary intake of fructose led to increased intestinal permeability and was associated with NAFLD onset [106]. The latter was further associated with increased levels of LBP, which were closely correlated with the degree of liver injury and with liver TNFA expression in patients with obesity [110]. More interestingly, oral treatment of patients having both insulin resistance and NASH with IgG-enhanced fraction of enterotoxic *E. coli* (ETEC) colostrum led to an improvement of insulin resistance and overall metabolic markers such as lowered lipid levels [148].

Leptin, an adipokine predominantly secreted by adipose tissue, enhances TNF-α production in LPS-stimulated Kupffer cells and its administration prompted elevated LPS-induced hepatic TNF-α production in wild-type rats. TNF-α in the liver of leptin-deficient Zucker rats, on the other hand, was unaltered after LPS stimulation [149], further accentuating a putative adipose tissue–liver crosstalk in endotoxemia-induced inflammation and metabolic impairment.

### 5.3. Pancreas

Translocation of bacterial and endotoxic bacterial components into the pancreas has also been related to changed function of this highly metabolic active tissue. In models of acute pancreatitis, impairment of small intestine mucosa evident from increased mucosal permeability to fluorescent latex microspheres and apical villi damage has been observed. Interestingly, labeled bacteria from the intestine were traced from the gut to mesenteric lymph nodes and to the pancreas, further pointing to a gut–pancreas inflammatory axis [150].

Although involvement of the pancreas in T2D has been thought of as a strictly endocrine functional loss of the organ, exocrine activity of the pancreas is paramount to normal digestive function and fat storage. Enterocyte-produced Angiopoietin-like 4 (Angptl4) is a potent inhibitor of gut luminal and pancreatic lipoprotein lipase (LPL) activity [151] and is inhibited by the gut microbiota. Germ-free mice display higher Angpt14 levels and lower pancreatic LPL activity, translated in reduced adipose tissue fat storage. Interestingly, HFD with coadministration of probiotic *Lactobacillus* strains led to reduced body fat alongside increased circulating levels of Angptl4, suggesting a relevant contribution of a specific microbial population to the expression of Angptl4 [21]. The endocrine pancreatic activity can similarly be influenced by microbiotal signals. An important observation in type 1 diabetes (T1D) is that inflammation of β-cells preceded seroconversion to autoantibody positivity [152]. Nonobese diabetic (NOD) mice were more prone to early onset and increased incidence of diabetes, when held under germ-free conditions [153]. This was accompanied by higher levels of cytokines, promoting an inflammatory state [154].

A direct modulation of pancreatic cell function has been mostly observed in experimental models, where LPS impaired insulin gene expression via TLR4-dependent NF-kB signaling [155].

### 5.4. Intestine

The intestine plays a pivotal role in T2D, and therapeutic approaches based on intestinal mechanisms have shown very convincing results related not only to weight loss but also overall cardiovascular health [156]. The intestine contributes to sensing energy and nutrient status and communicates with the brain via neuronal pathways and endocrine molecules to regulate energy homeostasis [157]. Moreover, the intestine harbors enteroendocrine active cells (L-cells). These cells produce and secrete GLP-1, GLP-2, and Peptide YY (PYY), which contribute to controlling appetite and regulate gut transit as well as ß-cell proliferation and insulin secretion in the pancreas [158]. Although germ-free mice have higher GLP-1 levels, this was not associated with improved glucose tolerance or insulin secretion. Considering the highest density of GLP-1-expressing L-cells is found in the colon [159] and that nutrients reach the colon much later than the insulin peak occurs, it is likely that colonic GLP-1 has another function. Specifically, it was shown that western diet in germ-free mice normalizes GLP-1 levels and accelerates gut transit, underscoring a role of GLP-1 in energy sensing, gut transit, and therefore energy exploitation of the small intestine [160], which could be regulated by the gut microbiota. Moreover, gut bacteria contribute to the production of secondary bile acids [161], which have been shown to signal through L-cells-expressed TGR5 in the intestine to increase GLP-1 secretion [162]. Bile diversion to the ileum (as seen in Roux-en-Y-gastric bypass) led to weight-independent improvement of glycaemia concomitantly associated with increased GLP-1 levels as well as levels of *Akkermansia muciniphila* in the gut [163].

Although GLP-1 receptor agonists show promising health effects in T2D, an idiosyncratic lack of response has been evidenced in some patients, suggesting GLP-1 resistance of unclear origins. Grasset et al. identified a set of bacteria in the ileum (Clostridiales, Bacteroidales, Burkholderiales, and TM7), which impaired the GLP-1-activated gut–brain axis, impacting gastric emptying and insulin secretion [164]. Moreover, treatment of subjects with insulin resistance and NASH with IgG-enhanced fraction of ETEC colostrum improved insulin secretion during oral glucose tolerance test (oGTT) in a GLP-1-dependent manner and reduced HbA1c levels and lipid profiles while increasing adiponectin levels [148].

In another study, microbially derived tryptophan metabolite indole led to an increase of GLP-1 production, which ceased after prolonged exposure to the metabolite, supporting the role of the microbiome in modulating L-cell function and therefore subsequently connecting host responses to the environment [165]. GLP-2, on the other hand, has been shown to improve gut epithelial function by improving barrier function and increasing epithelial cell regeneration [166]. GLP-2-agonists have subsequently been used to increase resorption surface in conditions such as short bowel syndrome, where they widely improve energy intake and symptoms in affected patients [167]. Prebiotic treatment of ob/ob mice reduced LPS levels and improved gut barrier, which was further associated with increased *Bifidobacteria* and *Lactobacillus* and was shown to be GLP-2-dependent [168]. However, the relationship between microbiota and glucose tolerance is not one-sided. Thaiss et al. have shown that hyperglycemia increases intestinal permeability via glucotoxicity by altering tight junction integrity and leads to an influx of microbial products into the systemic circulation and increased enteric infection dissemination driving the circulus vitiosus of hyperglycemia, intestinal barrier impairment, and inflammation [169].

### 5.5. Muscle

The average human body consists of approximately 42% and 36% of muscle in males and females, respectively. More importantly, insulin resistance in the skeletal muscle is considered the primary injury in T2D, observable decades before hyperglycemia and β-cell dysfunction were noted [170]. Subclinical inflammation in obesity has been associated with early insulin resistance brought upon by leptin-driven [149] and increased levels of TNF-α and subsequent decrease in insulin receptor substrate 1 (IRS-1) signaling and tyrosine kinase activity [171]. It is therefore possible that changes in the microbiome and subsequent inflammation can directly or indirectly affect muscle insulin sensitivity. Interestingly, early endotoxemia in the setting of infection has been associated with increased insulin sensitivity related to increased glucose uptake in the muscle, contributing to observed hypoglycemia in early sepsis [172]. In the disease progression of septic patients, hyperglycemia derived from increased insulin resistance becomes a common feature [173] and links between hyperglycemia and adverse outcomes in sepsis have been observed [174]. Hyperglycemia in endotoxemia is partly due to insulin resistance, as reflected by decreased glucose disposal rates, but also ensues from increased glycogen depletion in skeletal muscle and liver and decreased glycogen synthesis and glycogen synthase activity [175]. Extended periods of endotoxemia are characterized by a reduction of glucose utilization associated with nonoxidative glucose disposal impairment as well as increased levels of glucose and growth hormones [173], which are known for their insulin-antagonistic effects [176,177]. LPS treatment induced inflammation via increased expression of cytokines such as IL6, TNFA, IL1B, and PAI1 in adipose tissue, liver, as well as muscle, subsequently reducing muscle insulin action in HFD-fed [136], but also in wild-type, mice [178] and human myotubes [179]. It was furthermore associated with reduced oxidative capacity [180] as well as GLP-1 receptor-dependent modulation of changes in glucose metabolism [181]. In contrast, TLR4 mutation abolished LPS’ ability to stimulate the same cytokines, pointing to a pivotal role of the TLR4 signaling pathway [178,180]. This is further substantiated by observations of increased expression of TLR4 in skeletal muscle tissue in subjects with obesity and T2D [180,182]. Increased plasma LPS and LBP levels in subjects with obesity and T2D negatively correlated with muscle insulin sensitivity [179] as well as reduced incidence of heart disease and T2D in subjects with TLR4 gene polymorphisms [141,183].

### 5.6. Brain and Nervous System

The increased recognition of a putative gut–brain axis partly modulated by the gut microbiome and its links with metabolic, neurodegenerative [184], functional bowel disorders [185], as well as psychiatric disorders [186] has led to the discovery of several communication routes pertinent to interactions between host–environment interface and our central nervous system. Connecting pathways include gut hormone signaling to modulate appetite and gut motility, tryptophan metabolism and vagal nerve signaling, as well as SCFA effects on several tissues including adipose tissue to regulate the secretion of cytokines with central regulatory effects. The gut microbiome has moreover been suggested to influence behavior including feeding but also enteric inflammation leading to obesity, all of which have been reviewed in detail elsewhere [187]. The gut microbiome has proven essential for the development of the enteric nervous system by regulation of serotonin production and tryptophan metabolism as well as modulating 5-HT4R-specific signaling. Neuronal 5-Hydroxytryptophan furthermore acts as a suppressor of inflammation in the intestinal mucosa [188], whereas activation of 5-HT4R has similarly been shown to reduce inflammation in mice with colitis [189], suggesting a link between gut microbiota, enteric nervous system, inflammation, and subsequent bacterial translocation. Diseases dictated by central serotonergic activity arise later on and are associated with a decreased stability and diversity of the gut microbiome. These effects have been suggested to relate to the kynurenine pathway, leading to a reduction of tryptophan availability for central serotonin synthesis [190]. The interlinkedness of psyche and metabolism has been elegantly demonstrated in a study where microbiota from subjects with major depressive disorders was transferred into germ-free mice. These mice exhibited not only the depressive phenotype but also altered microbial metabolic networks as well as hippocampal metabolism characterized by a compromised carbohydrate and amino acid metabolism. These results suggest a mediational effect of microbiota-impacted central and peripheral metabolisms to induce depressive-like behaviours [191]. The vagal nervous system has similarly been shown to be involved in gut–brain modulation of metabolism. HFD in mice increased microbial acetate production in the gut, leading to an increased vagal stimulation, promoting glucose-stimulated insulin response and increased ghrelin secretion, resulting in a pathological feedback loop of hyperphagia and obesity [192]. Moreover, the use of artificial sweeteners such as saccharin was associated with the development of T2D and obesity, partly through induction of compositional and functional gut microbiota shifts in mice, which were associated with impaired production and secretion of GLP -1 and impaired glucose tolerance [193] as well as increased habituation to sweet taste in children [194]. Conversely, shifts in the gut microbiome brought on by prebiotic treatment have been suggested to amplify GLP-1 and PYY secretion [195]. The GLP1 receptor agonist’s effects on insulin secretion, gastric emptying, and subsequent improvement in glycaemia have been demonstrated to be dependent on vagus nerve activation and gut–brain axis recruitment [196]. The emergence of a bidirectional communication between the hypothalamic–pituitary–adrenal axis and the gut through increased permeability also underscores the relevance of stress, intestinal permeability, and inflammation in the regulation of host metabolism and health [197].

## 6. Bacterial Presence in Remote Tissues

Although the largest body of evidence has been put forward for the gut microbiome, many tissues accommodate adapted microbial consortia, which are finally accessible with culture-independent techniques. While several studies have focused on surrogate parameters or the evidence of bacterial components in the circulation to link intestinal permeability with metabolic disease, there is scarce research investigating the presence of bacterial components or bacteria in metabolically active tissues, their putative contribution to microenvironment changes and their relationship with tissue dysfunction and local inflammation. Moreover, little is known about tissue selectivity of bacteria, their source of origin, whether they are alive and occupying a relevant ecological niche, or whether there exist disease-specific extra-intestinal bacterial signatures (Table 2).

To this aspect, a limited number of studies have investigated the presence and composition of bacterial DNA in regard to its relationship with metabolic risk or disease in several tissues. While the evidence of the presence of bacteria in blood of even healthy individuals is accumulating [198,199], evidence for bacterial presence in other tissues such as liver, muscle, or fat depots has been controversial.

A first study reporting a link between circulatory bacterial load and metabolic disease was published in 2011. In a cohort of 3280 subjects followed for 9 years, 16S rRNA gene concentration was significantly increased in subjects who developed T2D over time [200] and was increased in subjects with abdominal obesity at follow-up. The bacterial load was predominantly related to Proteobacteria [200], which was an independent risk factor for cardiovascular disease development during follow-up [201]. The risk for T2D development has further been associated with specific taxa, with Bacteroides found to be protective against T2D and Sediminibacterium found to increase the risk for T2D [201]. Moreover, overt T2D was associated with higher detection rates of bacterial DNA in the blood [202], and subjects with bacterial translocation based on qPCR detection were less likely to experience resolution of T2D or significant improvement of insulin resistance and inflammation despite significant loss following bariatric surgery [111]. In subjects with liver cirrhosis, the circulating bacterial composition was similarly dominated by proteobacteria, which further echoes findings in healthy subjects and subjects with liver fibrosis [203,204], and associated with circulating inflammatory cytokine levels connecting circulating bacteria with systemic inflammation. Moreover bacterial composition was compartment-specific (central, peripheral, hepatic, and portal venous blood), supporting the notion of a tissue-specific compartmentalization [205].

The evidence for bacteria in other tissues related to the ominous octet has been practically nonexistent till of late. There is a large body of evidence for bacterial infections of the pancreas in acute pancreatitis but no data supporting contamination of the pancreas without acute inflammation. An exception is the recent evidence of oral bacteria in intraductal papillary mucinous neoplasms (IPMNs) preceding invasive pancreatic cancer [206]. Understanding effects of the (gut) environment on adipose tissue, a metabolically and inflammatory active organ and reservoir of lipids, is of growing importance, as underlined by the presence of environmental pollutants [207,208]. First proof for the transmucosal passage of bacteria has been provided by Amar et al., who could localize gavaged GFP-labeled *E. coli* in mesenteric adipose tissue of high-fat diet-fed mice. Similarly, Burcelin et al. have evidenced the presence of bacterial DNA in human adipose tissue [209]. In succession, few research groups have been able to validate these results [210], especially in humans. In 2016, Zulian et al. observed bacterial PCR products in isolated mature adipocytes from human adipose tissue, but sequencing revealed these products being attributed to *Clostridium histolyticum* only, which serves as a source for the collagenase used to isolate mature adipocytes [211]. Culturing experiments to validate these findings remained negative. In contrast, bacterial DNA was reported in mesenteric adipose tissue of 12 subjects with obesity belonging mostly to *Ralstonia* a year later [212]. Bacterial DNA was moreover detected in epicardial adipose tissue of subjects with acute coronary syndrome and stable angina, whereas none could be found in subjects with isolated mitral insufficiency [213]. The authors associated coronary heart disease with an increased susceptibility of the epicardial adipose tissue to bacterial colonization and inflammasome activation.

The lack of representative negative controls in these particular studies is the Achilles’ heel of all studies searching for compartment-specific bacterial signatures in human tissues, where negligible or very small amounts of bacterial DNA are expected.

Very recently, Anhê, Jensen et al. published data comparing the bacterial composition and load of liver, subcutaneous, visceral, and mesenteric adipose tissues as well as plasma samples. They noted the highest abundance for bacterial DNA in visceral adipose tissue and liver samples, whereas subcutaneous and mesenteric adipose tissue samples had similarly reduced amounts of bacterial DNA. Plasma samples did not contain significantly more bacterial DNA than negative controls. The authors were able to delineate preferential compartmentalization of eight specific genera in adipose tissue, whereas plasma samples differed in two tissue-specific genera. Interestingly, mesenteric adipose tissue displayed pronounced taxonomial differences as compared with other fat depots and an increased relative abundance of gut colonizers, consistent with the natural anatomical route of a presumed gut–liver axis. Whereas no differences in bacterial load were found within tissues between subjects with and without T2D, subjects without T2D displayed a significantly increased bacterial diversity in bacterial signature of mesenteric adipose tissue, pointing to a link between tissue-specific bacterial signature and glucose tolerance similar to observations of microbial diversity in gut microbiome studies. A specific strength of this study is the extensive inclusion of negative controls at each step of the preanalytical and experimental procedure accounting for operation field contamination at tissue collection, environmental contamination during tissue manipulation including air samples from surroundings and swab controls for used surfaces, as well as negative controls for DNA extraction, amplification, and sequencing, making it one of the first studies to present contamination-aware evidence of tissue-specific bacterial compartmentalization with a T2D extra-intestinal microbial signature, which was independent of obesity [214]. This evidence could further be expanded by recently published data from our group, where we succeeded in detecting adipose tissue borne living bacteria using catalyzed reporter deposition (CARD) - fluorescence *in situ* hybridization (FISH). We moreover quantified and sequenced 16S rRNA gene content in 75 patients with obesity and with or without T2D and could show that both bacterial quantity and taxonomy were associated with markers of inflammation and insulin resistance. This was further corroborated with functional tests in immortalized human subcutaneous preadipocytes where bacterial DNA challenge led to a bacterial DNA concentration-dependent stimulation of *TNFA* and Interleukin 6 (*IL6)* [215]. 

Evidence in this area has not been without controversy. Recently, Schierwagen et al. published a letter of response noting the important challenges to tackle when working with low-microbial-biomass samples [216]. Beyond the ones stated in the letter including low amounts of bacterial DNA, bacterial contamination from environment and material used, as well as high amounts of PCR inhibitors in human samples, we believe studies should go beyond experimental bacterial reduction and control to include rigorous bioinformatic steps to handle contaminating operational taxonomic units and taxa in downstream analyses. Although the jury is out on how best to tackle this issue, some suggestions from the community included—among others—completely discarding taxa seen in negative controls (on operational taxonomic unit (OTU) level), leading to a significant reduction in taxa possibly biologically relevant [217], but new more elegant methods taking distribution of taxa in negative controls on a frequency or prevalence basis have emerged as viable, more moderate alternatives [218]. In general, the importance for contamination reduction and control cannot be overstated. The appropriate computational approach to bioinformatically control for contamination is highly dependent on the environment sampled. A growing need to evaluate computational approaches prior to testing can be done using mock microbial communities in dilution series. There is indeed growing evidence for this kind of contamination control being necessary [216,217,219,220].

## 7. Conclusions

Several publications with converging lines of evidence support increased intestinal permeability and bacterial translocation as perpetrators in the development of metabolic disease. This evidence has linked obesity and T2D with altered qualitative and quantitative gut microbiome changes, increased permeability, and ensuing local inflammation in metabolically active remote organs as well as systemic inflammation accounting for an increased systemic insulin resistance. In the near future however, this area of research needs to move beyond association studies towards functional approaches to understand potential directionalities and mechanisms involved. Additionally, there is a need to elaborate hypotheses in preclinical and clinical settings including intervention studies, which actively modulate the gut microbiome and intestinal barrier to establish the relevance of gut permeability in human metabolism. The reason for this being that changes in the gut microbiome as well as intestinal permeability can both lead to inflammation, which itself can modulate these two adjacent axes, making establishing a clear sequence of events almost unmanageable.

In addition to bacteria, the intestine is also populated by other organisms such as archaea, yeast, and fungi as well as viruses, phages which can contribute similarly to host–microorganisms interactions [221,222,223]. The prevailing issue in current research is the lack of standardization in metagenomic procedures, making speedy major fundamental breakthroughs improbable but highly warranted when investigating the triangle of gut microbiome, intestinal permeability, and metabolic disease.

Moreover, there is a need to define “bacterial translocation”: whereas some authors equate increased intestinal permeability with bacterial translocation, others have used unreliable surrogate parameters, which reflect increased permeability to bacterial products such as LPS or host markers conferring a possible reaction to bacterial products. They all heavily depend on healthy subjects as a point of reference, making conveying absolute values and states of health and disease impossible, thereby impeding the standardization of the definition for “leaky gut” or bacterial translocation.

To this extent, the evidence of bacteria in the circulation and metabolically active organs should be the most direct proof for bacterial translocation. While the body of work in this area of research has been steadily increasing, with several works painting a coherent picture, it has not been without criticism, particularly in light of the controversy surrounding the presence of a placenta microbiome [219]. This criticism is based on several shortcomings in many studies, which include the lack of analytical controls to overcome contamination and the narrative approach, contributing little to elaborating the role of tissue microbiota in metabolic disease and developing mechanistic hypotheses and experimental work to test underlying pathways. To this end, we recommend thorough approaches including experimental designs accounting for contamination, technical biases, and errors as well as standardizing analytical approaches to include computational contaminant assessment and control [217]. This includes controlling for prelevement contamination as well as contamination ensuing from further downstream experimental work [217].

Recent developments including multitechnical approaches [224] and more recently, contaminant-aware approaches [214,217] to evidence the existence of extra-intestinal bacteria and their relationship with metabolism point to the fact that one cannot simply repudiate the existence of tissue-specific bacteria.

To this extent, the potential inherent to this field supports coordinated efforts to revisit many of the concepts introduced in the framework of this review. The aim would be to initiate clear, comprehensive, and nuanced approaches, which reflect our times and the immense development achieved in this area of research, in order to establish the clinical significance of these concepts and tap their therapeutic and preventative potential.

## Figures and Tables

**Table 1:** Markers of intestinal permeability in noncommunicable disease.

Permeability Marker	Tests	Direct/ Indirect	Sample Needed	Corresponding Literature
**Functional tests**				
**Lactulose/Mannitol**	Small intestinal permeability	direct	24 h Urine	Bosi et al., 2006 [130] Teixeira et al., 2012 [90] Genser et al., 2018 [92]
**lactulose/L-Rhamnoase**	Small intestinal permeability	direct	24 h Urine	Mooradian et al., 1986 [131] Wigg et al., 2001 [47] Wilbrink et al., 2019 [132]
**Chrom-51-Ethylen diamine tetraacetic acid** **(51 Cr-EDTA)**	Entire intestine permeability	direct	24 h Urine	Horton et al., 2012 [133]
**Circulating/fecal markers**				
**Zonulin**	Tight junction dysfunction	indirect	Serum/Plasma/Feces	Wang et al., 2000 [127] Moreno-Navarrete et al., 2012 [134] Zak-Gołąb et al., 2013 [135]
**Lipopolysaccharide (LPS)**	Endotoxemia	indirect	Serum/Plasma	Cani et al., 2007 [136] Damms-Machado et al., 2017 [89]
**Lipopolysaccharide Binding Protein ** **(LBP)**	Measurement via LPS Binding potential	indirect	Serum	Ruiz et al., 2007 [110] Ahmad et al., 2017 [94] Genser et al., 2018 [92]
**Calprotectin**	Gut inflammation	indirect	Serum Urin Plasma Feces	Ortega et al., 2012 [121] Pedersen et al., 2014 [137]
**Endotoxin core antibodies**	Endotoxemia	indirect	Plasma	Hawkesworth et al., 2013 [126]
**intestinal fatty acid binding protein (iFABP)**	Ischemia	indirect	Plasma/Serum	Cox et al., 2017 [109]
**Ex Vivo**				
**Ussing chambers**	Transepithelial electrical resistance (TEER)	direct	Intestinal biopsies	Genser et al., 2018 [92]

**Table 2:** Studies evidencing bacterial presence in remote organs and metabolic disease.

Reference	Study Population	Tissue	Detection Method	Findings	Limitations
Amar et al., 2011 [200]	3280; 3149 without diabetes, 131 with incident diabetes	Blood	16S rRNA gene concentration, pyrosequencing	16S concentration slightly higher in diabetes (0.13 vs. 0.15, *p* = 0.04) Adj. OR of incident diabetes for 1 SD 16S: 1.35 [1.1–1.6], *p* = 0.002, Proteobacteria dominant phylum	Not matched for sex, age Group size with incident diabetes small, no negative controls reported, DNA was air-dried
Amar et al., 2013 [201]	3936, with 3, 6, and 9 years follow-up (73 cardiovascular events)	Blood	16S rRNA gene quantification	Concentration of Proteobacteria was positively correlated with onset of cardiovascular events (OR 1.56 [1.1–2.2], *p* = 0.007)	Quantification of all bacteria (Eubac) was lower compared with Proteobacteria (Probac), Tertiles not equally distributed, no negative controls reported, DNA was air-dried
Burcelin et al., 2013 [209]	Not reported, Patients grouped by body mass index (BMI)	Adipose tissue stromal vascular fraction	16S rRNA gene pyrosequencing	Shift from Firmicutes to Proteobacteria with increasing BMI, Ralstonia was associated with BMI	Figure with previously unpublished data in Review article, no methods reported
Sato, Konazawa et al., 2014 [202]	100, 50 with T2D, 50 control subjects	Blood, fecal samples	Targeted 16S rRNA gene amplification using Yakult Intestinal Flora-SCAN with group-, genus- and species-specific primers	Gut bacteria associated with T2D found in fecal samples (i.e., *Lactobacillus*) were detected at sig. Higher levels in blood of T2D subjects (28% vs. 4%, *p* < 0.01)	No sequencing data, bias due to selection of primers, no negative controls reported
Ortiz et al., 2014 [111]	58 patients undergoing bariatric surgery and 3, 6, and 12 month follow-up	Blood	16S rRNA gene quantification, LPS measurement (Limulus amoeboyte lysate (LAL)-test)	Translocation rate at baseline: 32.8% After follow-up: 13.8% (3 month), 1.8% (6 month), 5.2% (12 month)	Follow-up does not distinguish between surgery procedure (Roux-en-Y-gastric bypass (RYGB) or sleeve gastrectomy (SG)), no control group, no negative controls reported
Païssé et al., 2016 [203]	30 healthy subjects	Whole blood, buffy coat, red blood cells, plasma	16S rRNA gene quantification, and sequencing of V3-V4 region by MiSeq	Most blood bacteria located in buffy coat (93.7%), followed by red blood cells (6.2%) and plasma (0.1%) Dominant phyla are Proteobacteria (~80%), Actinobacteria, Firmicutes, Baceroidetes	Small cohort size, No negative controls reported
Lelouvier et al., 2016 [204]	Discovery cohort with 50 patients and validation cohort with 71 patients, all obese but with different stages of liver fibrosis	Blood	16S rRNA gene quantification, and sequencing of V3-V4 region by MiSeq	Quantity of bacterial DNA increased in liver fibrosis, Actinobacteria decreased and Proteobacteria increased in liver fibrosis, Overall dominant phyla reported were Proteobacteria and Actinobacteria, Association between quantity and liver fibrosis but not bacterial taxa signature could be reproduced in validation cohort	16S metagenomic sequencing of stool was performed using different region (V1–V3), and sequencing platform (454 FLX), no negative controls reported, tissue in cohorts differed (buffy coat vs. whole blood) + large differences in quantification (652.6 vs. 3.1 copies/µL)
Pedicino et al., 2017 [213]	18 with acute coronary syndrome (ACS), 16 with stable angina (SA), and 13 controls from patients undergoing mitral insufficiency	Epicardial adipose tissue	16S rRNA gene amplification (V1–V3) and sequencing (n = 3 per group) on GS junior platform	Predominant species in ACS: Cyanobacteria Streptophyta and Proteobacteria Rickettsiale, in SA Proteobacteria Moracellaceae and Pseudomonas	No technical negative controls, only few samples sequenced
Udayappanet al., 2017 [212]	12 patients	Mesenteric-visceral adipose tissue	Denaturing gradient gel electrophoresis and Sanger sequencing	Bacteria were found in mesenteric tissue, Actinobacteria are dominant Gram-positive and *Ralstonia* Gram- negative bacteria. Fecal *R. picetti* increased in T2D	Small sample size, non-state-of-the-art method introduces bias in reported bacteria (cloning and Sanger sequencing instead of next-generation amplicon sequencing)
Schierwagen et al., 2018 [205]	7 patients with decompensated liver cirrhosis	Central, hepatic, peripheral, and portal venous blood (buffy coat)	16S rRNA sequencing	4 Phyla reported, dominated by proteobacteria and Actinobacteria, composition did not differ between compartments, *Pelomonas, Rahnella* among other genera correlated positively with inflammatory markers, *Esherchica and Salmonella* negatively	Limited methods reported due to format (Letter), small sample size, no control group
Anhê, Jensen et al., 2020 [214]	40 patients with obesity (20 without T2D, 20 with T2D)	Liver, blood, adipose tissue	16S rRNA quantification and sequencing (V3-4)	Bacterial DNA is present in adipose tissue and liver, Highest amounts were observed in liver an omental adipose tissue, diversity was highest in mesenteric adipose tissue, dominant phyla were Proteobacteria and Firmicutes	Although a strong point is negative controls, it becomes not clear how they were analyzed, clinical data is reported but not included in analysis
Massier, Chakaroun et al., 2020 [215]	75 patients with obesity (33 with T2D, 42 without T2D)	Omental, mesenteric, subcutaneous adipose tissue, blood	16S rRNA quantification and sequencing (V4-5) catalyzed reporter deposition - fluorescence in situ hybridization (CARD-FISH) bacterial DNA challenge in immortalized human preadipocytes	Bacterial DNA is present in all tested adipose tissue depots as well we blood, with dissimilarities between tissues being influenced by overall host inflammation and insulin resistance. Highest amounts of bacterial DNA were detected in the blood. Bacterial quantity was associated with macrophages infiltration and expression of inflammatory markers in adipose tissue. Living bacterial cells were detected in adipose tissue via CARD-FISH.	No inclusion of lean subjects
